# “Although Her Words Said No, Her Actions Spoke Otherwise”: Potential Jurors’ Understanding of the “Reasonable Belief” Element of Sexual Assault Law

**DOI:** 10.1177/10778012251323863

**Published:** 2025-03-12

**Authors:** Tessa Gillies-Goldsmith, Melanie A. Beres, Rachel Zajac

**Affiliations:** 1 2495 University of Otago, Otago, New Zealand

**Keywords:** reasonable belief, implied consent, sexual assault, rape, juror decision-making

## Abstract

Sexual assault laws in several jurisdictions require jurors to consider whether a defendant “reasonably believed” in consent. Using thematic analysis, we explored how potential jurors (*N* = 50) make judgments about consent communication and the behaviors that, when informed by the reasonable belief standard, are perceived to communicate (non)consent. Two themes captured the perception that consent is something that is implied, while non-consent is explicit. This narrative supports legal scholars’ concerns that the reasonable belief standard is applied in inappropriate ways—prioritizing a defendant's sense of implied consent over a complainant's attempt to refuse and failing to consider the defendant's honesty.

## Introduction

At the heart of every sexual assault and rape allegation is the notion of consent.^1^ Sex with consent can never be rape, and sex without consent always will be. Despite this seemingly clear distinction, consent to sex—and more specifically, the way people communicate consent and non-consent—is an issue that the courts have been trying to “get right” for decades (Quilter, 2020).

To understand how jurors might treat the issue of consent in court, we first need to understand how consent exists between partners more generally. A growing body of work has begun to establish what consent communication looks like in non-forensic situations. To date, this research has failed to support the legal assumption that people communicate clearly about whether they want or intend to have sex, despite the attention given toward clear communication in affirmative consent models (Beres, 2007; Beres & Farvid, 2010; Frith & Kitzinger, 1997; Hardesty et al., 2022; Hickman & Muehlenhard, 1999; Humphreys & Herold, 2007; Kitzinger & Frith, 1999; Newstrom et al., 2021; O’Bryne et al., 2008; Shumlich & Fisher, 2020).

When engaging in non-sexual discussion, people tend to be both direct and instant when accepting an offer from another person (e.g., “thanks, I'd love to come”; Kitzinger & Frith, 1999). When engaging in a refusal, however, they tend to be neither. Refusals are often characterized by delays, prefaces, and hedges (e.g., “uhh…” or “well…”), appreciations (“I’m so chuffed to be invited”), apologies (“I’m really sorry I can’t make it”), and justifications (“I’m just so busy with work right now”; Kitzinger & Frith, 1999). Furthermore, justifications are much more likely to comprise features that make the refuser unable*—*as opposed to unwilling—to accept the offer (“I have a work function that night”), thereby protecting the offer (and the person offering) from being seen as unwanted. This research suggests that people are often uncomfortable with explicit rejection and will use indirect means to “soften the blow.”

Research into consent for sexual activity shows us that this pattern holds true, suggesting that direct consent communication is not normative (Beres, 2007, 2010; Beres & Farvid, 2010; Frith & Kitzinger, 1997; Hardesty et al., 2022; Hickman & Muehlenhard, 1999; Humphreys & Herold, 2007; Kitzinger & Frith, 1999; Magnusson & Stevanovic, 2023; O’Bryne et al., 2008). For example, Beres (2010) identified the ambiguity of consent communication by asking adults aged between 19 and 30 years about communicating consent in casual sexual encounters. When describing how they would know that their partner was not interested in having sex, participants described a process of reading body language and picking up on cues that was not dissimilar to non-sexual communication. For example, they identified that if their partner froze up, pulled away, stopped kissing them, or showed any sign of discomfort, it would indicate an unwillingness to continue. Notably, participants did not indicate that they would talk to their partners about this behavior.

In a thematic analysis of young women discussing how they engage in sexual refusals, Kitzinger and Frith (1999) found that direct refusals were considered to violate social norms, a pattern also noted by Baldwin-White (2021) and Hardesty et al. (2022). Specifically, refusers were seen as rude, arrogant, or foolish, and it was considered abnormal to refuse a sexual advance without offering a reason (Kitzinger & Frith, 1999). Ambiguity, on the other hand, provided some social protection (Hardesty et al., 2022), especially when power dynamics are at play (Baldwin-White, 2021). Kitzinger and Frith's participants also reported that the reasons they gave were not the real reasons they did not want to have sex but were instead excuses that they felt their partners would find acceptable as to why they could not have sex (e.g., “I’ve got my period”). Moreover, participants identified that they often provided temporary refusals—a “not yet” instead of a “no.” Despite knowing that this tactic often led to further discomfort and social negotiation when the reason was no longer valid and the offer was made again, participants noted that this approach was more socially acceptable than an outright refusal. Participants agreed that it was important to soften the refusal with something that would avoid hurting the initiator's feelings (“it's not you, it's me”).

When someone is indirect in their refusal, they are at risk of that refusal not being received. More specifically, people's use of indirect strategies can result in attempts at rejection being misinterpreted, overlooked, or ignored (Beres, 2010; Lofgreen et al., 2021; Marcantonio et al., 2024). The miscommunication model—which suggests that ambiguous communication leads people to mistakenly assume consent—is often used by lay people in explaining where consent negotiation went wrong (Dardis et al., 2021; Frith & Kitzinger, 1997; Hansen et al., 2010). Research has also considered the role that desire might play in sexual refusals, in that the inherent motivator of desire may lead people to interpret signs of rejection differently to signs of agreement (Lofgreen et al., 2021; Marcantonio et al., 2024). However, researchers have also demonstrated that even when people can understand nonverbal cues, they might choose to interpret them differently. For example, Beres (2010) identified that young people can interpret nonverbal communication clues fairly well, suggesting that sexual advances that occur following an indirect refusal may sometimes reflect a person capitalizing on ambiguity in order to proceed.

Given the tendency for sexual refusals to be indirect, how do people expect to give consent to sex? The answer appears to vary widely—often an accumulation of specific behaviors engaged in over time (Hardesty et al., 2022). Some young adults believe they can “just know” when someone is interested in having sex with them, despite it never being verbally acknowledged (Beres, 2010, p. 6). Such knowledge appears to come from contextual factors (e.g., dancing at a club together and then going to either person's home), intimate physical behaviors (e.g., touching a person’s bottom or “getting it on” on the dancefloor), and interpreting the way a person looks at them or the way they “sync together” (Beres, 2010, p. 6). The presence of active participation in preintercourse intimacy is also considered informative—this might include pulling a partner closer, breathing heavily, touching a partner's body intimately, and kissing. Verbal acknowledgment that sex is about to occur is not guaranteed. People find asking for consent awkward (Shumlich & Fisher, 2020) and tend to see behaviors differently depending on who enacted them (e.g., self vs. partner; Newstrom et al., 2021).

Groggel and colleagues (2020) examined interviews with 83 college students, investigating how they expected consent to be communicated in a hypothetical scenario that did not include information on whether each party asked for or gave explicit consent to sex. Participants discussed how they might initiate sexual activity through indirect behaviors such as “giving them the eye” or “flirting, dancing, laughing.” The authors identified that participants would assign dual meanings to behaviors. For example, they recognized that inviting someone to your home after a party did not qualify as verbal consent, but it did indicate implied consent—a concept that participants frequently used to make sense of the dissonance that arose when consensual sex was thought to have occurred without explicit consent communication.

Taken together, these findings indicate that there is no universal way people communicate consent to sex. We can say, however, that direct verbal consent is not the norm and cannot be assumed to have occurred for sex to have been considered entirely consensual by all parties. Conversely, direct verbal refusal is not the norm and cannot be assumed to have occurred for sex to have been considered non-consensual. Given these findings, it would not be surprising that jurors struggle to make decisions about the presence of consent and criminal culpability.

When it comes to examining consent in the courtroom, numerous definitions of rape across jurisdictions hinge on whether a defendant's perception of consent was based on “reasonable grounds” (High, 2023; Oxford Pro Bono Publico, 2020). This element acts as a test of mental state: to be guilty of a crime, a defendant must have the required mental state to have criminal culpability, or *mens rea* (Gore, 2020). The reasonable belief standard refers to a legal defense to rape, which asks whether the defendant reasonably and honestly believed that the complainant was consenting—whether the complainant consented or not (Gore, 2020). If the defendant honestly believed they had consent to sex and this belief was reasonable, then they lacked the necessary *mens rea* to commit the crime (Gore, 2020). Due to the lack of corroborating evidence and eyewitnesses in sexual violence cases, the focus for decision-makers is instead placed on the complainant's actions and whether they appropriately demonstrated non-consent (Berliner, 1991; Burgin & Flynn, 2019). The defendant's mental state is inferred from the complainant's behavior; that is, would the complainant's actions lead a reasonable person to realize that consent had not been given?

There appears, then, to be a mismatch between how people expect to negotiate consent in their daily lives and the factors that jurors are expected to consider in court. On the one hand, research tells us that people endorse ambiguous nonverbal communication. Yet, when they enter a courtroom, jurors must identify behaviors as indicating a “yes” or a “no.” This dissonance was captured explicitly by Gray (2015), who asked 18 university students about acquaintance rape and the types of behavior—both verbal and non-verbal—that they thought were used to communicate consent. Participants identified behaviors that bear a resemblance to the findings of Beres (2007, 2010), Hickman and Muehlenhard (1999), and Frith and Kitzinger (1997) regarding non-verbal consent being normative. However, Gray's participants were quick to follow their descriptions of those behaviors with the disqualifier that they simply implied an interest in having sex but were not enough to communicate consent. As one participant put it, “It might say she may be interested, but it doesn’t say yes” (Gray, 2015, p. 343).

When it came to knowing when someone did *not* consent to sex, Gray’s participants differentiated themselves from ‘others’ who might overstep and assume consent was present based on those non-verbal behaviors. Participants claimed to understand that subtle behavior such as “pull[ing] back” could indicate a lack of consent from their partner (p. 345) and stated that they would stop appropriately. However, in the context of expressing their own non-consent, participants described verbal refusal as “the only way” women could be “clear” that they do not wish to have sex (p. 345). Furthermore, they described verbal refusal as being sufficient (“no should mean no”) as well as easy to do (“…you decide it's not for you, and then you say ‘no’”; p. 343). People who did not share these views were described as “guys who rape,” “rapists,” and someone who “wasn’t a nice guy” (p. 348).

Overall, Gray's participants described inconsistent preexisting beliefs. Participants were quick to reject the idea that they endorsed certain beliefs themselves (i.e., that nonverbal cues always constitute consent) and perceived people who did not possess the ability to respond to non-verbal consent cues as “rapists” (p. 348). Yet, in doing so, they endorsed the rape myth that regular “good guys” cannot rape. They also described multiple behaviors as indicating intent to have sex but then disqualified these behaviors from communicating actual consent—resulting in shifting conceptualizations of potentially misleading behavior. Finally, they described themselves as adept at registering non-verbal signs of non-consent, while simultaneously endorsing the notion that verbal rejection is the only way for a woman to reject a sexual advance clearly, and that such a rejection is straightforward to execute. This inconsistency might speak to the dissonance occurring when people's personal experiences and social knowledge do not align with laws asking for concrete evidence of (non)consent, and the resultant fear of being perceived as endorsing rape myths.

Given what we know about normal sexual communication, it is important to consider how people perceive the reasonable belief standard. Larcombe and colleagues (2016) investigated perceptions of the adequacy and scope of the reasonable belief standard when applied to sexual assault. The participants were four groups of sexual assault sector workers (*n* = 22), three groups of legal workers (*n* = 10), and four groups of community members (*n* = 29) in Victoria, Australia. Participants identified two main critiques of the reasonable belief standard itself. First, they identified that the standard “defines rape according to the perpetrator” as opposed to the victim, without requiring defendants to take any steps to be sure of their interpretation of events (Larcombe et al., 2016, p. 618). Participants noted that the lack of requirement to conduct any particular enquiry into consent to justify their belief allows the defendant to act recklessly or negligently, giving inappropriate priority to the perpetrator’s perspective.

Second, participants expressed concern that there was no consensus about whose definition of “reasonable” was being assessed. They identified that different people would have different conceptions of what is reasonable and that sexual offenders would likely think any belief they have is reasonable. When asked to apply the reasonable belief standard to a fictional case of alleged sexual assault, participants were entirely split on how the standard would frame behaviors from the case. The only consensus participants reached was that although there was no evidence for a belief in *affirmative* consent, reasonable belief in consent could not be unanimously ruled out, and a not guilty verdict was likely to be reached—despite general agreement that this was a case of rape.

Where does this leave consent communication under the reasonable belief standard? Research shows that normal sexual communication is often ambiguous and nonverbal (Beres, 2007; Beres & Farvid, 2010; Frith & Kitzinger, 1997; Hickman & Muehlenhard, 1999; Humphreys & Herold, 2007; Kitzinger & Frith, 1999; O’Bryne et al., 2008), yet the reasonable belief standard requires decision-makers to make binary inferences about a complainant's behavior (Gore, 2020). This mismatch appears to result in applications of the law that are ambiguous and hesitant at best (Gore, 2020) and inconsistent with consent law at worst (Chalmers & Leverick, 2018; Ellison & Munro, 2009, 2015; Finch & Munro, 2006). Those working in sexual assault-related fields recognize these issues (Larcombe et al., 2016), but it is crucial to investigate how potential jurors understand and apply the reasonable belief standard when using it to consider consent communication in a case of alleged sexual assault.

In the present study, we provided 72 adults with two vignettes depicting an alleged sexual assault and asked them to use the reasonable belief standard to guide their decision-making. We then used thematic analysis to explore (1) the way in which participants made judgments about consent communication, and (2) which behaviors—informed by the reasonable belief standard—were perceived to communicate (non)consent.

## Method

### Sample

We recruited 72 Amazon Mechanical Turk (MTurk) workers. Once data quality checks had been implemented and the data screened for poor-quality responses, the final sample comprised 50 participants. These participants ranged from 22 to 62 years in age (*M* = 39.1 years; *SD* = 10.06), and all resided in the United States. Thirty-five participants identified as white, five as Black or African American, five as Hispanic or Latino, two as Asian, and one as American Indian or Alaskan Native. Two participants did not provide race data. Thirty participants identified themselves as women and 20 as men. All participants reported that the primary language they spoke was English, although it was one participant's second language. The participants had varied educational qualifications (high school diploma *n* = 3, community college certificate *n* = 5, college education with no degree *n* = 10, bachelor's degree *n* = 25, master's degree *n* = 7).

### Materials

A questionnaire was constructed using Qualtrics and uploaded to Amazon MTurk. Consenting participants completed the questionnaire individually online. We employed two vignettes, each detailing a fictional sexual assault case (see Appendix). In Vignette A, protagonists Jamal and Katie met at a bar and returned to Jamal's apartment. Katie initially refused sex, saying she was too tired and drunk, but sex occurred later in the night. In Vignette B, protagonists Harry and Abigail matched on Tinder and saw each other at a house party. When they went upstairs, Harry made multiple attempts to engage in sexual intimacy which Abigail responded to with indirect statements about sex being unlikely to occur. They ended up having intercourse after a few minutes. The women in both vignettes make rape allegations a week later.

Different jurisdictions have varying levels of specificity regarding reasonable belief. Therefore, to guide their interpretation of the vignettes, participants were also provided with a statute for sexual assault in a country that has reasonable belief written into law: Aotearoa New Zealand (s128, New Zealand Crimes Act 1961). The reasonable belief standard for consent was defined for participants as follows:

The reasonable belief standard is a condition that means a person cannot be guilty of sexual assault if they believed on reasonable grounds that the victim was consenting, whether the victim was consenting or not. It means that if someone did not have consent for sex but made a reasonable mistake in thinking that they did, they would be found not guilty. However, if their belief in consent was not reasonable, they would be found guilty.

### Procedure

Vignettes were presented in counterbalanced order. After reading the first of two vignettes, participants gave their verdict and selected a reason for that verdict from a list of options. For a not guilty verdict, there were three options: no sex took place, sex took place but the complainant consented, or sex took place and the complainant did not consent but the defendant reasonably believed that she did. For a guilty verdict, there were two options: the defendant was aware that nonconsensual sex took place, or the defendant errantly believed that the sex was consensual and his belief was unreasonable.

After completing attention check questions about the vignette characters’ names, participants were asked to reflect on their decision-making and respond to the following short essay prompts:

1. In as much detail as you can, please explain why you reached the verdict that you did.
2. You chose [verdict reason] as your verdict reason. What led you to choose that reason, instead of [alternative verdict reason/s]?
3. Consider the reasonable belief standard. Please provide your own personal definition of what would constitute a reasonable belief in consent and what would constitute an unreasonable belief in consent. You may give examples of behavior if this helps.

Participants were then presented with the second of the two vignettes and asked the following questions:

1. Imagine you were asked to make a judgment on this case about whether the defendant's belief in consent was reasonable. What judgment would you make and why?
2. What else would you want to know from the complainant or the defendant in order to make your judgment easier?
3. Overall, what do you think about the reasonable belief standard for sexual assault? Is this an appropriate way of judging consent?

Finally, participants were asked whether they had any other thoughts or comments about the two cases, the reasonable belief standard, or their decision-making process. They then provided demographic information before being thanked and redirected to a new webpage to receive their compensation.

### Analysis

We used a combined approach to thematic analysis (Braun & Clarke, 2006) to analyze the data set. The primary researcher first became familiar with the data through the process of transferring it from Qualtrics into document form and then reading the data set multiple times. She then coded the transcripts through an empathetic lens (i.e., taking words at face value), identifying emerging ideas and making note of all potential codes as they arose. Next, she used theoretical knowledge to categorize these ideas and make further interpretations using a suspicious lens (i.e., considering hidden meaning; Willig, 2013), before creating a coding scheme and re-analyzing the data set applying the scheme to the data.

Another research team member then read through the dataset and applied the coding scheme; researchers discussed the outcome to ensure the data had not been misrepresented and to support consensus across the research team. The primary researcher then organized the codes into interpretive categories that linked them meaningfully and produced insight into the perceived relationships between them, reviewing and revising them multiple times to produce an explanatory framework for answering the research question.

## Results and Discussion

Thirteen participants found the defendants guilty (*n* = 9 thought the defendant was aware that non-consensual sex took place; *n* = 4 thought the defendant errantly believed that the sex was consensual and his belief was unreasonable) and 37 participants found the defendants not guilty (*n* = 5 thought sex took place but the complainant consented; *n* = 32 thought sex took place and the complainant did not consent but the defendant reasonably believed that she did).

The following section combines the data and their interpretation to form an integrated analysis and discussion about how participants made judgments about consent communication and the behaviors that, when informed by the reasonable belief standard, were perceived to communicate (non)consent. Two themes were identified: *Consent is Implied* and *Non-Consent is Explicit*.

These two themes captured the idea expressed throughout the dataset that consent is something that is implied, while non-consent is explicit. The number and range of behaviors that were seen to imply consent were vast, but all shared the understanding that typical sexual communication is unspoken. In comparison, the behaviors seen as required to communicate nonconsent were specific, explicit, and regulated by the participants. The inclusion of consent behaviors and exclusion of non-consent behaviors, and the transition from one to the other, created a narrative in which consent is implied to be the default setting of people everywhere at any moment.

### Consent Is Implied

Given the number of individual occurrences that communicated such a large number of specific behaviors, a single excerpt cannot illustrate the breadth of this category. Instead, we briefly present a list to illustrate this point before returning to analysis. The following behaviors were identified as giving the defendant reasonable belief in the complainant's consent to sex: flirting, kissing and making out, laughing, swiping right on the defendant on Tinder, leaving a public place with the defendant, going home with the defendant, being in the defendant's apartment, going upstairs with the defendant, going to a bedroom with the defendant, lying on a bed with the defendant, playing hard-to-get, allowing one's clothes to be removed, touching the defendant, staying at the defendant's house overnight, having a “*generally positive reception*,” and any reciprocation or engagement in behaviors while in bed with the defendant. Staying at the location of the assault afterward, having a positive demeanor afterward, and waiting a week to report the assault were also seen to strengthen the defendant's reasonable belief, despite occurring after the belief about consent had been formed.

This list is important because it illustrates the baseline we refer to when we say that consent is the default. Not only were these behaviors seen to indicate a reasonable belief in consent, but they, at times, also spoke to the presence of consent itself. They rejected the notion of consent being an ongoing process and effectively removed the character's ability to retract consent once a lower-level intimate behavior had occurred. Participant 43 (woman, 42, Hispanic, college education, no degree) said: “She consented by going home with him. She consented by allowing him to touch her for a long time. She consented by getting in his bed and sleeping with him.” When asked if there was any information that would make their judgment on the case easier, Participant 38 (woman, 50, American Indian or Alaska Native, bachelor's degree) said: “Nothing, they are both adults, it wasn’t violent, it was consensual or she wouldn’t have been in his bed, drunk.” Participant 16 (man, 33, White, bachelor's degree) expressed a similar point of view, stating:

The fact that a woman is willingly laying in bed with another man, not fighting back, and simply saying a few hard-to-get lines is not a lack of consent. If she didn’t consent, she’d jump out of bed and leave. Or at the very least fight back so there would be evidence.

In courtrooms, *equivocal conduct* is a term used to refer to behavior that has a double sense (Cavallaro, 1996). On the part of the complainant, it is behavior that could be considered misleading to the defendant, such as ambiguous behavior or submitting out of fear (Berliner, 1991; Cavallaro, 1996). Both Berliner (1991) and Cavallaro (1996) identified cases in which this term has been applied in judicial decisions. Such behavior included willingly accompanying a man to his apartment, having a conversation, failing to continue resisting after initially digging fingernails into the accused's wrist, and not escaping a car at a stop sign—behaviors similar to those identified by our participants as indicating consent. It appears that potential jurors are likely to pick up on behaviors that could be considered ambiguous and incorporate them into their definition of a reasonable belief. Carline and Gunby (2011) interviewed 14 barristers who also referenced a series of non-sexual behaviors as amounting to evidence of a reasonable belief; these included flirting, showing signs of attraction, and going to a bedroom together. Lofgreen et al. (2021) found that men conflate what they perceive to be signs of sexual desire with sexual consent, sometimes to such a degree that participants did not distinguish these as two separate judgments. Burgin and Flynn (2019) argued that this creates an implied consent narrative that allows defendants to use any behavior that was not outright resistance as ambiguous, and possibly implying consent.

Participants also expressed that engaging in these “misleading” behaviors was enough to override a verbal statement of non-consent. For example, Participant 41 (woman, 57, White, master's degree) said: “Although Abigail's words said no, her actions spoke otherwise.” Participant 28 (man, 47, White, bachelor's degree) said: “She had said no earlier to sex. But, her actions after she went to bed make it seem like she was okay.” And Participant 44 (man, 31, White, bachelor's degree) said:

In this situation, you could argue that because Katie explicitly stated that she wasn’t interested in having sex with Jamal that this would be considered rape. However, because she engaged and reciprocated while in his bed, it completely vitiates that claim.

The participants acknowledged that both complainants had rejected sex with the defendants, yet they believed certain behaviors made that verbal communication redundant. These excerpts indicate that participants place a high value on misleading behavior in forming their definition of a reasonable belief.

This finding is consistent with Beres (2010), who found that people routinely engage in numerous behaviors that they believe give the impression of being interested in sex. The finding is also consistent with Gray (2015), who found that mock jurors identify these behaviors as relevant to consent communication. However, Beres’ participants stated that these behaviors were only indicative of interest, and not consent itself. Gray's participants similarly distanced themselves from such endorsement but claimed that other, less enlightened jurors might interpret the behaviors as indicating actual consent. Our participants explicitly stated that these behaviors supported a reasonable belief in consent, spoke to consent itself through implicit consent, and were powerful enough to override explicit non-consent communication. It is of note, however, that neither Beres’ (2010) study nor Gray's (2015) study required participants to render verdicts on the scenarios discussed, raising the possibility that our mock jurors’ interpretations of nonverbal behaviors might have been influenced by efforts to justify the verdicts they reached (Kunda, 1990; Nickerson, 1998).

### Non-Consent Is Explicit

#### Verbal Rejection

Although consent was seen to be implied through a large number of behaviors gradually increasing in intimacy, non-consent was seen to be something that was stated or acted explicitly. Many of our participants saw a verbal rejection of the defendant's advances as sufficient and necessary to judge the defendant's belief in consent as unreasonable. When explaining their thinking about Vignette A, Participant 25 (man, 36, Black or African American, college education, no degree) said:

In this case I would actually say this was not a reasonable assumption. A clear no was given earlier in the night. With a clear no already established getting verbal consent should be key before attempting intercourse again and that did not happen….

Participant 27 (woman, 42, Hispanic or Latino, bachelor's degree) agreed, saying

…in this case the victim says clearly she didn’t want to have sex because she was too tired and drunk. This was a clear NO, even though she was in his apartment and they were having a good time.

Verbal communication was also highlighted in Vignette B, in which Abigail's rejections were less direct—shutting down a joke about them being about to have sex and telling Harry that he was being “cheeky” while stopping him from removing her top. Participant 34 (man, 38, Asian, master's degree) identified that

Abigail had verbally communicated multiple times through both her words and actions that she did not want to go all the way… I think this would constitute as an unreasonable assumption of consent to have sex on Harry's part.

Participant 42 (woman, 38, White, bachelor's degree) agreed, saying that,

On more than one occasion Abigail mentioned that she was not interested in having sex with him…When his friend made a comment about Harry being up next Abigail mentioned that she wasn’t interested. When they were in the room and Harry was fondling her she made it clear that she was a bit uncomfortable by telling him that he is a bit frisky.

These participants perceived Abigail's remarks to be clear enough to void consent.

However, this indirect communication was not always perceived as sufficient. Participant 30 (man, 45, White, community college certificate) said,

sure he was constantly pressuring for this but at any point she could have put her foot down but did not. Although she did not say yes, she did not say no either. For rape there must be an explicit no, and at no point did was there an explicit no.

Participant 25 (man, 36, Black or African American, college education, no degree) attempted to sympathize with the complainant: “I can understand sometimes people have different or weird reactions to what is happening…,” but ultimately rejected her account; “the fact that she didn’t stop him, or didn’t say NO I don’t want to have sex, can be interpreted by anybody that she was okay with the intercourse.” Despite seeming aware of possible responses to trauma (i.e., freezing, appeasing the aggressor, dissociating; Mason & Lodrick, 2013), Participant 25 fell back on messages of victim responsibility (Payne et al., 1999). Participant 25 further specified how Abigail's attempts to reject Harry would not have registered with him:

I’m all for No means no…But playfully addressing Harry's friend is not the same as addressing Harry. At no point did she let him know for sure NO and was even still being playful with him after telling him that he was getting a little carried away.

Here the participant removes themselves even further from their initial trauma-informed statement and prioritizes the perspective of the defendant—Harry was unable to know Abigail's true feelings because she failed to address him in a way that would have registered.

Mentions of non-consent were frequently paired with an adjective that disqualified verbal statements that did not reach a required level. Examples included, “an explicit no,” “clearly expressed,” “a clear no,” and a “genuine attempt.” Participant 44 (man, 31, White, bachelor's degree) described Abigail's communication as being, “pillow talk and playing coy” which “doesn’t exactly qualify.” Participant 27 (woman, 42, Hispanic or Latino, bachelor's degree) said she personally avoids any misconceptions about her intention by saying, “if I want to flirt with someone but I don’t want to have sex then I would clearly say that I don’t want to have sex.” Her response indicates that the level of directness expected or required to make verbal communication effective is very high. It was clear that participants considered verbal communication the most important thing the complainant could do. Yet, they consistently rejected the complainants’ attempts at verbal communication for being too indirect, unclear, and disingenuous.

Participant 2 (woman, 46, White, master's degree) demonstrated the significance of explicit verbal communication when saying, “I can see why he might get the idea that she wanted to have sex with him, except that she explicitly said, I don’t want to have sex.” This excerpt seems to insinuate that without the explicit statement, the defendant had every reason to believe he had consent and that this belief could be derived entirely from non-verbal information. In fact, the complainant's agency was almost secondary to these external factors; if she had not spoken aloud and explicitly, then consent would have been assumed. As referenced earlier, Participant 27 (Woman, 42, Hispanic or Latino, Bachelor's degree) also hinted at the assumption that consent was the default, indicating that it was a woman's responsibility to correct this assumption, saying,

In my case as a woman if I don’t want to have sex with someone I would avoid any situation where the other person would think I want sex. And also, if I want to flirt with someone but I don’t want to have sex then I would clearly say that I don’t want to have sex.

Participant 49 (woman, 62, undisclosed race, bachelor's degree) put it more bluntly: “I feel the absence of words or actions indicates consent.” It appears that explicit rejection is necessary to denounce the assumed truth that consent is always there unless stated—explicitly, clearly, and toward the defendant—otherwise.

These requirements are inconsistent with literature showing that sexual refusals are indirect and can be vague (Beres, 2007, 2010; Kitzinger & Frith, 1999). In Vignette B, Abigail used a palliative to reject Harry's attempt at taking off her top, a common technique used to soften non-sexual refusals (Kitzinger & Frith, 1999). Participant 25 (Man, 36, Black or African American, College education, no degree) interpreted her soft refusal as a “*not yet”*—another common refusal technique that avoids immediate discomfort but risks future re-proposals (Kitzinger & Frith, 1999). In Vignette A, Katie provides an account for why she cannot have sex as opposed to stating that she does not want to have sex. This account left an opening for the defendant to assume consent should those factors no longer be considered valid. For example, Participant 39 (Woman, 34, Black or African American, Bachelor's degree), “assume[ed] Jamal thought she got some sleep and the alcohol had worn off.” Our complainants used common refusal techniques, yet these techniques reduced the perceived validity of their refusals. Our participants appeared to hold a standard for verbal rejections above and beyond what is typical in sexual encounters.

#### Physical Resistance

Non-consent can also be communicated through physical resistance, another highly explicit form of communication. Participant 16 (man, 33, White, bachelor's degree) described, “fighting back, screaming, recording a withdrawal of consent. There has to be clear physical material evidence that consent wasn’t given*.”* Participant 38 (Woman, 50, American Indian or Alaska Native, Bachelor's degree) described an unreasonable belief as being where the complainant,

… fought him off, punched and kicked him in the nut sack and he still forced himself on her, that would be rape… the reasonable belief should be that any adult gives consent for sex if they [don’t] open their mouth and state no, push the person away, or kick them in the vag or balls.

The use of force was also referenced by Participant 43 (woman, 42, Hispanic or Latino, college education, no degree), who said: “If what she said was even remotely true, she would have high-tailed it out of there immediately or gave a detailed reason on why she could not, like he held her hostage or something.”

Interestingly, these participants described an unreasonable belief in consent as involving force or violence. Instead, such behaviors should eliminate *any* belief that the complainant was consenting to sex and would therefore indicate that the defendant intentionally committed or tried to commit sexual assault. Put simply, if a partner is kicking and screaming or you have had to hold them hostage, it is unlikely that you are still under the honest impression that they might want to have sex with you. Our participants were asked to define what a reasonable belief in consent meant to them; for many, it meant a lack of physical resistance.

This finding indicates that a reasonable belief is being used as a proxy for the outdated physical resistance standard (Berliner, 1991). When the physical resistance standard was removed from many American jurisdictions’ definition of rape, there was concern that there would be an increase in the risk of miscarriages of justice against those wrongfully accused (Berliner, 1991). Berliner argued that this fear was informed by two beliefs: first, that the accused will be convicted solely on the victim's subjective sense of violation, even if their behavior indicated consent; and second, that a reasonable person will assume that a lack of physical resistance means the victim is consenting. In an attempt to replace the objectivity of the physical resistance standard and to strengthen the subjectivity of the honest belief standard, courts have used reasonable belief as a form of objective reflection of a person's subjective experience (Berliner, 1991; Burgin & Flynn, 2019).

Berliner argued that the complainant's internal state of consent would be communicated through acting like someone who gave consent and that these actions would give the defendant reason to believe it is a consensual encounter. It should then follow that the defendant's behavior would be seen as an objective reflection of their internal state of believing that they had consent. They would be evaluated on whether they acted like someone who intended to gain consent for sexual activity, and the outcome of these actions would give them a reason to believe they had consent.

In court, however, this logic is only applied to the complainant (Berliner, 1991; Burgin & Flynn, 2019; Ewing, 2017). The defendant's behavior is not considered an objective measure of their intent. Instead, legal emphasis is misplaced such that both parties’ subjective states are evaluated solely on the complainant's behavior. If the complainant's actions did not sufficiently communicate their internal state of non-consent, then the defendant is not found to have criminal intent (Berliner, 1991; Burgin & Flynn, 2019; Ewing, 2017). We arrive back at the premise of physical resistance; if the complainant did not physically resist the defendant's advances, then the defendant could have had reasonable belief in their consent and, therefore, cannot have had criminal intent.

By requiring physical resistance to be present, malicious intent has been equated with unreasonable belief. In fact, Participant 7 (Woman, 38, White, Bachelor's degree) stated explicitly that, “reasonable belief would be if there was no malicious intent… I would consider reasonable belief when someone makes honest mistake or misunderstanding.” Participants offer no consideration of an honest, yet still unreasonable, belief, speaking to legal scholars’ concerns that there is an inappropriate collapse of the two elements of a reasonable belief (Berliner, 1991; Burgin & Flynn, 2019).

### Implications

The above analysis highlights that implied consent—through nonverbal communication and an absence of physical resistance or verbal rejection—might play a significant role in how jurors make judgments about consent communication and what behaviors are perceived to communicate (non)consent. Most concerningly, these behaviors spoke to the presence of consent itself and overruled verbal non-consent. Participants in our sample used implicit consent to define what they considered to be a reasonable belief, resulting in very little consideration being given to the defendant's behavior and mental state. In other words, they considered what any defendant might infer from the complainants’ behavior, not whether the defendants in our vignettes honestly believed they had consent.

Legal scholars have argued that the reasonable belief standard is in danger of being applied in such a way that the two essential elements—the subjective component of whether a belief is honestly held and the objective component of what is reasonable—are improperly collapsed (Berliner, 1991; Burgin & Flynn, 2019; Cossins, 2019; Elvin, 2008; Gore, 2020). Our findings lend support to this argument—participants considered the nature of assumed and implied consent to lead to a situation where any reasonable person would likely have developed the same belief as the defendant, while ignoring the subjective element of honestly held belief.

These concerns can be a reality in courtrooms. For example, in the case of *R v Ewanchuk* (1999), the trial judge accepted that the complainant said “no” multiple times, yet he found her conduct to have “implied consent.” This stance allowed the defendant to claim he had a reasonable belief in consent and he was acquitted—a ruling which was then upheld by the Court of Appeal. The case was brought to the Supreme Court who ruled that a defense of implied consent did not, in fact, exist in Canadian law and the acquittal was overturned.

This approach taken in *R v Ewanchuk* (1999) is most concerning when we think about cases where there might be cause to find reasonable context but the defendant in question did not possess an honest belief. For example, coercion is a common element of unwanted sexual experiences (Pugh & Becker, 2018). A defendant who used coercion and pressure to intimidate a partner into agreeing to sex would be able to use her words to testify that his belief in her consent was reasonable. If the complainant did not dispute those words, there would be cause to describe the defendant's belief in consent as reasonable.

Our findings clearly showed the presence of rape myths in mock jurors’ reasoning about sexual assault cases, supporting previous research (Ellison & Munro, 2009, 2010; Leverick, 2020; Süssenbach et al., 2017). Despite some recent research finding that people try to reject rape myths when discussing sexual assault in focus groups (Gray, 2015; Larcombe et al., 2016), our results indicated that these beliefs are still likely to be carried into the courtroom in some form. Lawyers can highlight these misconceptions to bolster (in the case of the prosecution) or challenge (in the case of the defense) the complainant's credibility and the defendant's blameworthiness (Zydervelt et al., 2017).

Some of our findings are likely to add to complainants’ concerns when making the decision whether to report sexual assault to authorities (Orth, 2002; Patterson, 2011; Sable et al., 2006; Weiss, 2010), but there is nonetheless some reason for optimism. For example, we found that participants expected defendants to do more to ensure their interpretation of consent is correct in spaces where sex might be seen as less ‘expected.’ These findings suggest that perpetrators cannot always rely on expectations about verbal refusal to protect them from being seen as responsible (O’Bryne et al., 2008), despite knowing that non-verbal consent is the norm (Beres, 2007, 2010; Frith & Kitzinger, 1997).

These findings also have implications for sexual assault prevention. Research shows that responses to sexual communication vary more as a function of the situation than as a function of an individual's proclivity toward sexual violence (Lofgreen et al., 2021). Our findings support the idea that men who do not intend to assault their partners are at risk of doing so. Lofgreen and colleagues (2021) suggested that conflating desire with consent may act as the mechanism for over-reliance on implied consent behavior. We would agree that sexual assault prevention programs might benefit from educating young men on the limits of making assumptions based on their internal state of desire in response to their partner's behavior.

At the time of writing, sexual violence prevention advocates around the world are calling for further law reform regarding definitions of consent. For example, a petition was presented to the Parliament of Aotearoa New Zealand in 2022, calling for legislation that would provide a clear affirmative definition of consent and recognize the need for free and voluntary agreement at the time of the sexual act. Although the petition did not go so far as to call for a clear definition of reasonable belief, having a definition of what consent is—rather than simply what it is not—is a step in the right direction. In 2021, New South Wales Australia adopted an affirmative stance whereby consent is not to be assumed and a defendant is required to have taken steps to confirm that consent has been granted. Our findings would call for clear guidance being provided to decision-makers to support their understanding of nonverbal consent communication—in particular, to understand that although consent is often nonverbal, so is nonconsent.

### Limitations

This research is potentially limited by the fact that American participants were asked to apply an Aotearoa New Zealand law. Americans may have different general understandings of law or attitudes regarding sexual assault that could make it hard to apply a law that does not align with their preexisting beliefs. For example, Americans living in a state with stricter definitions of consent might hold stronger rape myth endorsement (Fakunmoju et al., 2021), which could have resulted in their responses reflecting more rape myth endorsement and less critical thinking about the reasonable belief element. However, research suggests that American samples are not unique in their potential endorsement of sexually coercive behaviors and that these beliefs also exist in Aotearoa New Zealand (Tapara, 2017). Given that sexual assault laws differ from state to state in the USA (see DeMatteo et al., 2015), finding a sample that had the same baseline expectations for the law would have resulted in a narrow set of perspectives. An attempt was made to mitigate this limitation through clear instructions for participants to use the law that was explicitly provided, as opposed to their own understanding of rape and its prosecution.

It is prudent to note that our choice of defendant names could have exerted a biasing effect on participants—namely, Jamal could have been perceived as a person of color and therefore vulnerable to more punitive judgments (Mitchell et al., 2005, but see, for example, Fraser et al., 2023). We cannot say with any certainty that this was the case as we did not analyze the vignettes separately. However, given our research questions focused on how potential jurors approached their task, regardless of their verdict, we consider this limitation to be a relatively minor one.

With the relative recency of MTurk as a data collection method, empirical research into possible limitations of the site is in its infancy. Many of the concerns raised about MTurk participants relate to studies using classic cognitive tasks that rely on the technological quality of the worker's computer (e.g., response time studies), or tasks where workers’ honesty cannot be guaranteed (e.g., tasks invalidated by online searches for correct answers; Crump et al., 2013). The present study avoided such concerns by asking workers for their subjective opinions in a simple survey response format. One possible avenue for future research includes using an alternative online recruitment method (such as *Prolific*) that has a greater variety of potential samples, so data collection is not limited to the United States. Notably, this site allows for individual country selection, meaning that researchers in Aotearoa New Zealand can conduct research with more applicability to their home country and its laws.

Research into juror decision-making has unique validity challenges (Breau & Brook, 2007; Kerr & Bray, 2005). Factors that are commonly highlighted as validity issues, yet are relatively flexible when designing a study, include the sample (e.g., university samples, adults from the community), the research setting (e.g., online, a psychology lab, a real courtroom), how the trial materials are presented (e.g., live re-enactment, video, written vignette), which trial elements are included (e.g., deliberation), and the dependent variables (e.g., dichotomous versus continuous measures of guilt, sentence length; see Bornstein, 1999 for a review). Although we are confident that our chosen methodology of having an adult community sample review written case material is likely to produce comparable results to other methods (Bornstein, 1999), true ecological validity cannot be demonstrated until these findings are replicated in naturally occurring representative circumstances (Breau & Brook, 2007). Researchers employing simulated jury trials, regardless of methodology, will always struggle to mimic the stakes associated with a real jury's task (Breau & Brook, 2007; but see Ellison & Munro, 2009, 2010, for a potential solution).

## Conclusion

Sexual consent communication research has shown us that nonverbal consent is the norm and direct rejections are rare (Beres, 2007; Beres & Farvid, 2010; Frith & Kitzinger, 1997; Hickman & Muehlenhard, 1999; Humphreys & Herold, 2007; Kitzinger & Frith, 1999; O’Bryne et al., 2008), yet the laws that jurors use to make decisions about sexual assault require them to make black and white judgments based on vague information (Gore, 2020). The reasonable belief standard especially directs jurors to use the complainant's behavior to infer the defendant's mental state (Berliner, 1991; Burgin & Flynn, 2019). Our research demonstrates that, in the eyes of potential jurors, consent can be implied by any number of indirect and ambiguous behaviors, while the behaviors seen to communicate non-consent are specific, explicit, and regulated by the participants. This focus on the behavior of the complainant with little inquiry into the honesty of the defendant represents an improper collapse of the two elements of the reasonable belief standard, whereby implied consent is prioritized—even over verbal non-consent.

## Notes

1. Sexual assault is a heavily gendered issue. The majority of perpetrators are cisgender men and the majority of victims are non-men (Smith et al., 2018). Although anyone can be a victim or perpetrator of sexual assault, rape myths arose from—and perpetuate—existing power structures in which men dominate and oppress women (Berliner, 1991). When considering trials relating to sexual assault, then, we are—by and large—considering issues of consent leading to men being charged with sexual assault against women.

## Appendix

### Vignettes

*Vignette* A. Katie (28) and Jamal (32) meet at a local bar. Katie came from work with some colleagues. Jamal is with a group of friends who have been there for some time and are quite rowdy. Katie makes eye contact with Jamal a number of times and smiles at him. At some stage, Jamal leaves the group and comes up to Katie. They talk for some time and Jamal buys her drinks, they are affectionate and laugh often. As the night goes on, they leave the bar and start to kiss outside. Jamal asks Katie, “Do you want to come back to mine then?” and she accepts.

At Jamal's apartment, they listen to music and have a few more drinks. They continue kissing, and Jamal begins to make more sexual advances toward Katie by touching her breasts under her dress and putting his hand down her underwear. Katie moans and tells Jamal to “keep going”. At Jamal's suggestion they move to the bedroom and this activity continues on the bed. After about 5 min, Katie goes to the bathroom. When she returns, Jamal gets a condom out of his bedside drawer. Katie says to him, “I don’t want to have sex sorry, I am too tired and drunk”. Jamal puts the condom away and they get into bed in the spoon position, with Jamal behind Katie.

Later in the night, Jamal feels Katie wriggle her butt against his crotch. He starts touching her again and she moans. While kissing her, Jamal rolls Katie over, unbuttons her dress, removes her underwear, and begins penetration. This continues until Jamal orgasms and they fall asleep. A week later, Katie arrives at her local police station and reports Jamal for rape. The following paragraphs detail Jamal's and then Katie's description of events.

Jamal was brought to the police station the following day to record a statement giving his side of the story. Jamal remembered Katie and acknowledged that she spent the night at his apartment the previous week. According to Jamal, the sex between him and Katie was entirely consensual. He recalled Katie initially saying she didn’t want to have sex, which he accepted. However, he said that she later changed her mind and started pressing her buttocks against his crotch in bed and moaning. He said he began to reciprocate and unbuttoned her dress and that Katie then lay on her back and opened her legs. Jamal said that he then removed her underwear and initiated sexual intercourse. He reported that neither of them said anything before he began penetration but that they were kissing so that was expected. Jamal stated that nothing about the sex suggested Katie wasn’t consenting, and that on the contrary she was a willing participant.

Katie stated that Jamal began to touch her in the night but that she was half asleep and did not know what was happening, so she tried to feign sleep noises. When he began to unbutton her dress, Katie said she froze. Unsure what to do, she said she allowed herself to be rolled onto her back and Jamal took off her underwear. Katie said that it happened so fast that she did not know how to slow things down and that she remained unresponsive to him throughout the encounter. The next morning, Katie said she did not know what to say, and that she was hungover and confused. She got dressed and left Jamal's apartment at about 9am.

*Vignette B*. Abigail (20) and Harry (24) are students at university, Abigail studies Nursing while Harry is completing a science major. They have some mutual friends and have matched with each other on Tinder, resulting in Harry sending Abigail a message but she did not respond. One night at a party, Abigail and Harry start talking. They spent most of the evening together, drinking and flirting—with Harry leaning close to Abigail and putting his arm around her, and Abigail kissing him on the cheek after teasing him.

Toward the end of the night, Abigail and Harry go upstairs. They both stumble as they climb the staircase and hold onto each other laughing. As they approach the bedroom another couple emerges, they are flushed and adjusting their clothing. The boy fist-bumps Harry as they pass each other and says, “You’re up dude,” to Harry. Abigail says “Hey I don’t think so!” and laughs. Once they enter the bedroom Harry locks the door and joins Abigail on the bed.

They begin to kiss and touch each other. Harry goes to pull off Abigail's top and she says “Hey now, don't go getting any ideas”. Harry laughs and her top stays on. They kiss some more, with the intensity increasing. Harry reaches under Abigail's skirt and rubs her genitals, over her underwear. Abigail laughs and says “Someone's being cheeky”. This continues for a minute or two. Harry then stands and pulls Abigail toward the edge of the bed, rolling her onto her stomach. He then lifts her skirt and kisses her butt cheeks while undoing his trousers, Abigail laughs and wriggles. Harry then inserts his penis into Abigail's vagina and penetrates her for a few minutes, before adjusting positions and rolling Abigail onto her back again. At one point during the encounter, Harry says “You like that huh” to which Abigail does not reply. Harry orgasms and they both adjust their clothes. Harry then pulls Abigail upright and kisses her, before they both return to the party downstairs. A week later, Abigail goes to their university's sexual assault support service. They then call the police and report Harry for rape. The following paragraphs detail Harry's and then Abigail's description of events.

Harry is called in for questioning later that day. Harry confirms that he and Abigail had sex but is adamant that the encounter was consensual. He describes Abigail flirting heavily with him and said he thought her responses to his friend fist-bumping him and him trying to take her top off was just to “play coy”. He said that he thought she was enjoying him touching her genitals and was not telling him to stop, so he continued. Harry said that she did not say anything when he rolled her over or unbuttoned his trousers and that he assumed her lack of response to his statement was simply because she was enjoying herself and her heightened breath spoke for itself. Harry said that she was happy and flirty with him immediately following the sex and that she stayed at the party for hours afterwards and seemed in good spirits.

Abigail stated that she spent the night flirting with Harry and that she agreed to go upstairs. However, she claimed that she did not want to go “all the way” and that she tried to make that clear by rejecting Harry's friend's assumption and then gently rebuffing him when he tried to undress her. When Harry began touching her genitals Abigail said that she began to feel uncomfortable at his persistence, but felt embarrassed about rejecting his advances again. When Harry rolled her over and kissed her bottom, Abigail said that she thought he was playfully ending their encounter and that they would return to the party. She said that she did not notice he had undone his trousers until she felt him begin to penetrate her. At this point, Abigail said that was shocked and didn't understand what had just happened. She described Harry as “vigorously” thrusting while she closed her eyes and waited for it to be over. When Harry was finished, Abigail said she did not know how to react and so pretended everything was normal. She returned to the party and kept drinking to try and forget about what had happened, but avoided Harry for the rest of the night.
